# Supplementary material for: The Systems Biology Research Tool: evolvable open-source software
Source: BMC Syst Biol. 2008 Jun 29;2:55. doi: 10.1186/1752-0509-2-55 (PMC2446383; doi:10.1186/1752-0509-2-55)
Supplement: Additional file 1 — SBRT Archive. An archive of the current version of the Systems Biology Research Tool. [file 1752-0509-2-55-S1.zip › sbrt-1.4.0/doc/developers_guide/api/sbrt/shell/io/class-use/FileFormat.html]

Uses of Class sbrt.shell.io.FileFormat


|  |  |  |
| --- | --- | --- |
| **Overview** \| **Package** \| **Class** \| **Use** \| **Tree** \| **Deprecated** \| **Index** \| **Help** | |  |
| PREV   NEXT | **FRAMES**    **NO FRAMES**     **All Classes** |  |


---


## **Uses of Class sbrt.shell.io.FileFormat**

| Packages that use FileFormat | |
| --- | --- |
| **sbrt.shell.io** | Provides classes and interfaces for reading and writing files. |
| **sbrt.shell.io.fba** | Provides classes and interfaces for reading and writing files relevant to Flux Balance Analysis. |
| **sbrt.shell.mng** | Provides classes and interfaces for creating process managers. |
| **sbrt.shell.mng.fba** | Provides classes and interfaces for creating Flux Balance Analysis process managers. |
| **sbrt.shell.text** | Provides classes and interfaces for defining formats for basic objects. |

Uses of FileFormat in sbrt.shell.io

| Fields in sbrt.shell.io declared as FileFormat | |
| --- | --- |
| `static FileFormat` | `FileFormat.GZIP_SER`             Used to denote a file containing serialized objects that has been compressed using the GNU program gzip. |
| `static FileFormat` | `FileFormat.GZIP_TEXT`             Used to denote a plain-text file that has been compressed using the GNU program gzip. |
| `static FileFormat` | `FileFormat.SERIALIZED`             Used to denote a file containing serialized objects. |
| `static FileFormat` | `FileFormat.TEXT`             Used to denote a plain-text file. |

| Methods in sbrt.shell.io that return FileFormat | |
| --- | --- |
| `FileFormat` | `SerializedOutputFile.getFileFormat()`             Returns the format of this file. |
| `FileFormat` | `RomoFileSource.getFileFormat()`             Returns the format used to create files of type `RomoFile`. |
| `FileFormat` | `ComoFileSource.getFileFormat()`             Returns the format used to create files of type `ComoFile`. |
| `FileFormat` | `TextInputFile.getFileFormat()`             Returns the format of this file. |
| `FileFormat` | `SmoFileSource.getFileFormat()`             Returns the format used to create files of type `SmoFile`. |
| `FileFormat` | `RomiFileSource.getFileFormat()`             Returns the format used to create files of type `RomiFile`. |
| `FileFormat` | `ComiFileSource.getFileFormat()`             Returns the format used to create files of type `ComiFile`. |
| `FileFormat` | `SerializedInputFile.getFileFormat()`             Returns the format of this file. |

| Methods in sbrt.shell.io with parameters of type FileFormat | |
| --- | --- |
| `static TextOutputFile<java.lang.String>` | `TextOutputFile.getBasic(java.lang.String fileName, FileFormat fileFormat)`             Returns a basic text output file with the provided file format. |
| `static TextInputFile<java.lang.String>` | `TextInputFile.getBasic(java.lang.String fileName, FileFormat fileFormat)`             Returns a basic text input file. |
| `static boolean` | `SerializedOutputFile.isValidFormat(FileFormat fileFormat)`             Indicates if the provided file format is valid for a serialized output file. |
| `static boolean` | `TextOutputFile.isValidFormat(FileFormat fileFormat)`             Indicates if the provided file format is valid for a text output file. |
| `static boolean` | `SerializedFileIterator.isValidFormat(FileFormat fileFormat)`             Indicates if the provided file format is valid for this type of file iterator. |
| `static boolean` | `TextFileLineIterator.isValidFormat(FileFormat fileFormat)`             Indicates if the provided file format is valid for this type of file line iterator. |
| `static boolean` | `TextInputFile.isValidFormat(FileFormat fileFormat)`             Indicates if the provided file format is valid for a text input file. |
| `static boolean` | `SerializedInputFile.isValidFormat(FileFormat fileFormat)`             Indicates if the provided file format is valid for a text input file. |

Constructors in sbrt.shell.io with parameters of type FileFormat

`AbstractMapInputFile(java.lang.String fileName,
FileFormat fileFormat,
MapFormat<K,V> mapFormat)`
  
          Constructs a new map input file from the provided arguments.

`AbstractMapInputFile(java.lang.String fileName,
FileFormat fileFormat,
java.util.Set<? extends K> requiredKeys,
MapFormat<K,V> mapFormat)`
  
          Constructs a new map input file from the provided arguments.

`AbstractMapOutputFile(java.lang.String fileName,
FileFormat fileFormat,
MapFormat<K,V> mapFormat)`
  
          Constructs a new vector output file from the provided
arguments.

`AbstractMapOutputFile(java.lang.String fileName,
FileFormat fileFormat,
java.util.Set<? extends K> keys,
MapFormat<K,V> mapFormat)`
  
          Constructs a new map output file from the provided
arguments.

`AbstractMapOutputFile(java.lang.String fileName,
FileFormat fileFormat,
java.lang.String allVariables,
MapFormat<K,V> mapFormat)`
  
          Constructs a new map output file from the provided
arguments.

`ComiFile(java.lang.String fileName,
FileFormat fileFormat,
MapFormat<K,V> mapFormat)`
  
          Constructs a new row-oriented map input file from the
provided arguments.

`ComiFile(java.lang.String fileName,
FileFormat fileFormat,
java.util.Set<? extends K> requiredKeys,
MapFormat<K,V> mapFormat)`
  
          Constructs a new row-oriented map input file from the
provided arguments.

`ComiFileSource(java.util.Iterator<java.lang.String> fileNameSource,
FileFormat fileFormat,
MapFormat<K,V> mapFormat)`
  
          Constructs a new source of column-oriented map input files.

`ComiFileSource(java.util.Iterator<java.lang.String> fileNameSource,
FileFormat fileFormat,
java.util.Set<? extends K> requiredKeys,
MapFormat<K,V> mapFormat)`
  
          Constructs a new source of column-oriented map input files.

`ComoFile(java.lang.String fileName,
FileFormat fileFormat,
java.util.Set<? extends K> columnHeaderVariables,
MapFormat<K,V> vectorFormat)`
  
          Constructs a new column-oriented map output file
from the provided arguments.

`ComoFile(java.lang.String fileName,
FileFormat fileFormat,
java.lang.String allColumnHeaderVariables,
MapFormat<K,V> vectorFormat)`
  
          Constructs a new column-oriented vector output file
from the provided arguments.

`ComoFileSource(java.util.Iterator<java.lang.String> fileNameSource,
FileFormat fileFormat,
MapFormat<K,V> mapFormat)`
  
          Constructs a new source of column-oriented map output files.

`ComoFileSource(java.util.Iterator<java.lang.String> fileNameSource,
FileFormat fileFormat,
java.util.Set<? extends K> keys,
MapFormat<K,V> mapFormat)`
  
          Constructs a new source of column-oriented map output files.

`ComoFileSource(java.util.Iterator<java.lang.String> fileNameSource,
FileFormat fileFormat,
java.lang.String keys,
MapFormat<K,V> mapFormat)`
  
          Constructs a new source of column-oriented map output files.

`RomiFile(java.lang.String fileName,
FileFormat fileFormat,
MapFormat<K,V> mapFormat)`
  
          Constructs a new row-oriented map input file from the provided arguments.

`RomiFile(java.lang.String fileName,
FileFormat fileFormat,
java.util.Set<? extends K> requiredKeys,
MapFormat<K,V> mapFormat)`
  
          Constructs a new row-oriented map input file from the provided arguments.

`RomiFileSource(java.util.Iterator<java.lang.String> fileNameSource,
FileFormat fileFormat,
MapFormat<K,V> mapFormat)`
  
          Constructs a new source of row-oriented map input files.

`RomiFileSource(java.util.Iterator<java.lang.String> fileNameSource,
FileFormat fileFormat,
java.util.Set<? extends K> requiredKeys,
MapFormat<K,V> mapFormat)`
  
          Constructs a new source of row-oriented map input files.

`RomoFile(java.lang.String fileName,
FileFormat fileFormat,
MapFormat<K,V> vectorFormat)`
  
          Constructs a new row-oriented vector output file from the
provided arguments.

`RomoFile(java.lang.String fileName,
FileFormat fileFormat,
java.util.Set<? extends K> rowHeaderVariables,
MapFormat<K,V> mapFormat)`
  
          Constructs a new row-oriented map output file
from the provided arguments.

`RomoFile(java.lang.String fileName,
FileFormat fileFormat,
java.lang.String allRowHeaderVariables,
MapFormat<K,V> vectorFormat)`
  
          Constructs a new row-oriented vector output file
from the provided arguments.

`RomoFileSource(java.util.Iterator<java.lang.String> fileNameSource,
FileFormat fileFormat,
MapFormat<K,V> mapFormat)`
  
          Constructs a new source of row-oriented map output files.

`RomoFileSource(java.util.Iterator<java.lang.String> fileNameSource,
FileFormat fileFormat,
java.util.Set<? extends K> keys,
MapFormat<K,V> mapFormat)`
  
          Constructs a new source of row-oriented map output files.

`RomoFileSource(java.util.Iterator<java.lang.String> fileNameSource,
FileFormat fileFormat,
java.lang.String keys,
MapFormat<K,V> mapFormat)`
  
          Constructs a new source of row-oriented map output files.

`SerializedInputFile(java.lang.String fileName,
FileFormat fileFormat)`
  
          Constructs a new text input file using the provided arguments.

`SerializedMapInputFile(java.lang.String fileName,
FileFormat fileFormat)`
  
          Constructs a new input file using the provided arguments.

`SerializedMapInputFile(java.lang.String fileName,
FileFormat fileFormat,
java.util.Set<? extends K> requiredKeys)`
  
          Constructs a new input file using the provided arguments.

`SerializedOutputFile(java.lang.String fileName,
FileFormat fileFormat)`
  
          Constructs a new serialized output file from the provided
arguments.

`SmoFile(java.lang.String fileName,
FileFormat fileFormat,
java.util.Set<? extends K> allKeys)`
  
          Constructs a new serialized map output file from the
provided arguments.

`SmoFileSource(java.util.Iterator<java.lang.String> fileNameSource,
FileFormat fileFormat,
java.util.Set<? extends K> keys)`
  
          Constructs a new source of serialized map output files.

`TextInputFile(java.lang.String fileName,
FileFormat fileFormat,
Parser<E> fileLineFormat)`
  
          Constructs a new text input file using the provided arguments.

`TextInputFile(java.lang.String fileName,
FileFormat fileFormat,
Parser<E> fileLineFormat,
java.util.Collection<java.lang.Character> commentCharacters)`
  
          Constructs a new text input file using the provided arguments.

`TextOutputFile(java.lang.String fileName,
FileFormat fileFormat,
Formatter<T> lineFormat)`
  
          Constructs a new text output file using the provided
arguments.

Uses of FileFormat in sbrt.shell.io.fba

| Methods in sbrt.shell.io.fba that return FileFormat | |
| --- | --- |
| `FileFormat` | `SfoofSource.getFileFormat()`             Returns the file format used to create files of type `SerFbaOptOutputFile`. |
| `FileFormat` | `RofoofSource.getFileFormat()`             Returns the file format used to create files of type `RowOrFbaOptOutputFile`. |
| `FileFormat` | `CofoofSource.getFileFormat()`             Returns the file format used to create files of type `ColumnOrFbaOptOutputFile`. |

Constructors in sbrt.shell.io.fba with parameters of type FileFormat

`CofoofSource(java.util.Iterator<java.lang.String> fileNameSource,
FileFormat fileFormat,
java.util.Set<java.lang.String> columnHeaders,
FbaOptFileLineFormat fbaFormat)`
  
          Constructs a new source of column-oriented FBA optimization output files.

`CofoofSource(java.util.Iterator<java.lang.String> fileNameSource,
java.lang.String fileNameSourceName,
FileFormat fileFormat,
java.util.Set<java.lang.String> columnHeaders,
FbaOptFileLineFormat fbaFormat)`
  
          Constructs a new source of column-oriented FBA optimization output files.

`ColumnOrFbaOptOutputFile(java.lang.String fileName,
FileFormat fileFormat,
java.util.Set<java.lang.String> columnHeaders,
FbaOptFileLineFormat fbaFormat)`
  
          Constructs a new column-oriented FBA optimization output file.

`FluxIntervalInputFile(java.lang.String fileName,
FileFormat fileFormat,
java.util.Set<LinearComb<java.lang.String>> requiredVariables,
MapFormat<LinearComb<java.lang.String>,Interval> mapFormat)`
  
          Constructs a new flux-interval input file.

`RofoofSource(java.util.Iterator<java.lang.String> fileNameSource,
FileFormat fileFormat,
java.util.Set<java.lang.String> rowHeaders,
FbaOptFileLineFormat fbaFormat)`
  
          Constructs a new source of row-oriented
FBA optimization output files.

`RofoofSource(java.util.Iterator<java.lang.String> fileNameSource,
java.lang.String fileNameSourceName,
FileFormat fileFormat,
java.util.Set<java.lang.String> rowHeaders,
FbaOptFileLineFormat fbaFormat)`
  
          Constructs a new source of row-oriented
FBA optimization output files.

`RowOrFbaOptOutputFile(java.lang.String fileName,
FileFormat fileFormat,
FbaOptFileLineFormat fbaFormat)`
  
          Constructs a new row-oriented FBA optimization output file.

`RowOrFbaOptOutputFile(java.lang.String fileName,
FileFormat fileFormat,
java.util.Set<java.lang.String> rowHeaders,
FbaOptFileLineFormat fbaFormat)`
  
          Constructs a new row-oriented FBA optimization output file.

`SerFbaOptOutputFile(java.lang.String fileName,
FileFormat fileFormat,
java.util.Set<java.lang.String> filterKeys,
FbaOptFileLineFormat fbaFormat)`
  
          Constructs a new serialized FBA optimization output file.

`SfoofSource(java.util.Iterator<java.lang.String> fileNameSource,
FileFormat fileFormat,
java.util.Set<java.lang.String> filterKeys,
FbaOptFileLineFormat fbaFormat)`
  
          Constructs a new source of serialized
FBA optimization output files.

`SfoofSource(java.util.Iterator<java.lang.String> fileNameSource,
java.lang.String fileNameSourceName,
FileFormat fileFormat,
java.util.Set<java.lang.String> filterKeys,
FbaOptFileLineFormat fbaFormat)`
  
          Constructs a new source of serialized
FBA optimization output files.

Uses of FileFormat in sbrt.shell.mng

| Methods in sbrt.shell.mng that return FileFormat | |
| --- | --- |
| `static FileFormat` | `AbstractProcessManager.getFileFormat(ManagerInput<java.lang.String,java.lang.String> input)`             Returns the parsed value for `AbstractProcessManager.FILE_FORMAT` in the provided input. |
| `static FileFormat` | `AbstractProcessManager.getFileFormat(java.lang.String formatName)`             Parses and returns the file format corresponding to the provided string. |
| `static FileFormat` | `AbstractProcessManager.getTextFileFormat(ManagerInput<java.lang.String,java.lang.String> input)`             Returns the parsed value for `AbstractProcessManager.FILE_FORMAT` in the provided input. |
| `static FileFormat` | `AbstractProcessManager.getTextFileFormat(java.lang.String fileFormatString)`             Returns the text file format corresponding to the provided string. |

Uses of FileFormat in sbrt.shell.mng.fba

| Methods in sbrt.shell.mng.fba with parameters of type FileFormat | |
| --- | --- |
| `static MapInputFile<java.lang.String,java.lang.Double>` | `FluxActivityManager.getFluxVectorFile(java.lang.String fileName, FileFormat format, Fluxome fluxome)`             Returns the flux vector input file with the provided name. |
| `static TextInputFile<LinearComb<java.lang.String>>` | `ObjectiveFunctionManager.getInputFile(java.lang.String fileName, FileFormat format, Fluxome fluxome)`             Returns the input file with the provided name. |
| `static MapOutputFile<java.lang.String,java.lang.Double>` | `FbaManager.getMultiFluxVectorOutputFile(java.lang.String fileName, FileFormat fileFormat, java.util.Set<java.lang.String> rxnNameHeaders, Fluxome fluxome)`             Returns an empty output file that is suitable for storing multiple flux vectors. |
| `static FbaOptOutputFile` | `FbaOptManager.getMultiResultsOutputFile(java.lang.String fileName, FileFormat fileFormat, java.util.Set<java.lang.String> dataHeaders, Fluxome fluxome)`             Returns an empty output file that is suitable for storing multiple FBA optimization results. |
| `static TextOutputFile<LinearComb<java.lang.String>>` | `RandomObjectiveManager.getOutputFile(java.lang.String fileName, FileFormat format, java.io.PrintWriter out, IrrevRxnFluxome fluxome)`             Returns an empty output file that is suitable for storing randomly generated objective functions. |
| `static MapTextOutputFile<java.lang.String,Interval>` | `RandomConstraintsManager.getOutputFile(java.lang.String fileName, FileFormat format, java.util.Set<java.lang.String> headers, IrrevRxnFluxome fluxome)`             Returns an empty output file that is suitable for storing multiple sets of flux constraints. |
| `static MapOutputFile<java.lang.String,java.lang.Double>` | `FbaManager.getSingleFluxVectorOutputFile(java.lang.String fileName, FileFormat fileFormat, java.util.Set<java.lang.String> rxnNameHeaders, Fluxome fluxome)`             Returns an empty output file that is suitable for storing a single flux vector. |
| `static FbaOptOutputFile` | `FbaOptManager.getSingleResultOutputFile(java.lang.String fileName, FileFormat fileFormat, java.util.Set<java.lang.String> dataHeaders, Fluxome fluxome)`             Returns an empty output file that is suitable for storing a single FBA optimization result. |

Uses of FileFormat in sbrt.shell.text

| Methods in sbrt.shell.text that return FileFormat | |
| --- | --- |
| `FileFormat` | `FileFormatFormat.parse(java.lang.String source)`             Parses the provided string and returns its corresponding file format. |

| Methods in sbrt.shell.text with parameters of type FileFormat | |
| --- | --- |
| `java.lang.String` | `FileFormatFormat.format(FileFormat fileFormat)`             Returns a formatted string representation of the provided file format. |

---


|  |  |  |
| --- | --- | --- |
| **Overview** \| **Package** \| **Class** \| **Use** \| **Tree** \| **Deprecated** \| **Index** \| **Help** | |  |
| PREV   NEXT | **FRAMES**    **NO FRAMES**     **All Classes** |  |


---
